# Molecular Imprinting Applications in Forensic Science

**DOI:** 10.3390/s17040691

**Published:** 2017-03-28

**Authors:** Erkut Yılmaz, Bora Garipcan, Hirak K. Patra, Lokman Uzun

**Affiliations:** 1 Department of Biotechnology and Molecular Biology, Aksaray University, 68100 Aksaray, Turkey; yilmazerkut@yandex.com; 2 Institute of Biomedical Engineering, Bogazici University, 34684 Istanbul, Turkey; bgaripcan@gmail.com; 3 Department of Clinical and Experimental Medicine, Linkoping University, 58225 Linköping, Sweden; hirak.kumar.patra@liu.se; 4 Department of Chemistry, Hacettepe University, 06381 Ankara, Turkey

**Keywords:** molecular imprinting, forensic science, toxicology, analytical methods, pre-concentration

## Abstract

Producing molecular imprinting-based materials has received increasing attention due to recognition selectivity, stability, cast effectiveness, and ease of production in various forms for a wide range of applications. The molecular imprinting technique has a variety of applications in the areas of the food industry, environmental monitoring, and medicine for diverse purposes like sample pretreatment, sensing, and separation/purification. A versatile usage, stability and recognition capabilities also make them perfect candidates for use in forensic sciences. Forensic science is a demanding area and there is a growing interest in molecularly imprinted polymers (MIPs) in this field. In this review, recent molecular imprinting applications in the related areas of forensic sciences are discussed while considering the literature of last two decades. Not only direct forensic applications but also studies of possible forensic value were taken into account like illicit drugs, banned sport drugs, effective toxins and chemical warfare agents in a review of over 100 articles. The literature was classified according to targets, material shapes, production strategies, detection method, and instrumentation. We aimed to summarize the current applications of MIPs in forensic science and put forth a projection of their potential uses as promising alternatives for benchmark competitors.

## 1. Introduction

The area of forensic science emerged due to the need for scientific techniques for investigating and proving crimes. Forensic science is quite a multidisciplinary area of study with ten and more subdivisions like chemistry, biology, toxicology, geology, archeology, anthropology, astronomy, engineering, etc. All these subdivisions have different methods for problem solving and use a series of specialized tools. In general, “problem solving” in the area of forensic analysis have two meanings: First, identifying the physical evidence or a questioned sample, and the second one is comparing the results with a known material to find the origin of the questioned sample. Molecular imprinting techniques present solutions for both of these requirements as well. Due to their versatility, molecular imprinted polymers (MIPs) have gained many applications in a variety of areas [1]. Versatile usage, stability and recognition capabilities make them a perfect candidate for the use in forensic sciences [2]. MIPs can be prepared in different physical shape and sizes while conferring them with some multi-functional smart material capabilities, like magnetic, stimuli-responsive, fluorescence labelling, etc. These functions support many possible application areas in the field of forensic sciences.

## 2. A Brief Theory of Molecular Imprinting

**Figure 1:**
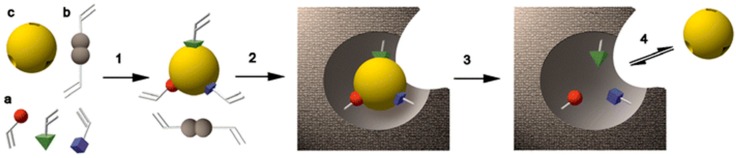
The molecular imprinting principle. a: functional monomers; b: cross-linker; c: template molecule; 1: assembly of the prepolymerisation complex; 2: polymerization; 3: extraction; 4: rebinding. Reprinted with permission from [5].

Molecular imprinting is the method of producing tailor-made complementary cavities against a targeted structure called the template. By means of these smart and complementary cavities, the resulting molecules have both chemical and physical recognition capabilities; therefore, they are also classified as biomimetic receptors or plastic antibodies [3]. Molecular imprinting is a method for producing selective binding sites in highly cross-linked synthetic polymeric matrices [4]. It is generally achieved via the self-assembly of functional monomers around a “template” and then polymerization of these pre-polymer complexes in the presence of extensive crosslinkers, which is only one of the generally followed synthetic routes and is called non-covalent imprinting. These monomers and ratio in the pre-polymer complex are chosen according to their affinity towards the template. After the template removal from the polymeric matrix, the imprinted cavities come out with both chemical and physical recognition capabilities (Figure 1) [5].

**Figure 2:**
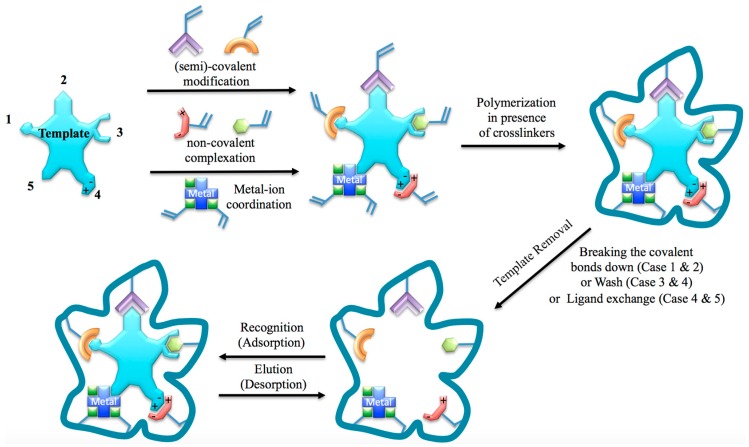
Schematic representation of five main types of interactions used for molecular imprinting purpose. 1: covalent; 2: semi-covalent; 3: covalent; 4: ionic; 5: metal ion coordination.

In imprinting history, researchers have integrated several polymerization techniques into the imprinting process which has resulted in five main types of molecular imprinting techniques. These can be summarized as covalent, non-covalent, semi-covalent, ionic, and metal coordination methods [2]. A schematic representation of these molecular imprinting techniques is shown in Figure 2.

Each of these techniques has certain advantages with respect to the affinity, selectivity, kinetics and reproducibility of the final polymers.

## 3. Imprinting Approaches for Forensic Science

In this review, we have focused our attention on compiling the imprinting literature for forensic science applications after briefly summarizing the history of imprinted polymers. In this context, we have clustered the studies into groups related to target structures, material shapes, production strategy, application method and detection platforms. In each subsection, we summarize the related studies while mentioning its novelty, contribution and importance for forensic science applications.

### 3.1. Target Structures

Availability of molecular imprinting as an analytical tool for variety of target structures is advantageous on forensic chemistry and forensic toxicology. Some of target molecules related to forensic sciences were summarized below:

*Legal/illicit Drugs:* There is a wide range of abused legal or illicit drugs that come across in forensic cases. Some of the most common drugs are given below with the examples from the molecular imprinting literature. Pain relievers, cough/cold medicines, prescription sedatives like benzodiazepines, and barbiturate sleeping aid medicines are the most studied drugs [6,7,8,9]. Ariffin et al. reported the extraction of diazepam and other benzodiazepines from hair samples. They reported a high recovery up to 93% with a good precision (RSD = 1.5%) and a limit of detection (LOD) and quantification (LOQ) of 0.09 and 0.14 ng/mg, respectively [6]. Anderson et al. compared the benzodiazepine extraction performance of an imprinted solid-phase extraction (SPE) system for 10-post-mortem scalp hair samples. The samples were chosen as blood samples of drug-related deaths with a positive benzodiazepine result. They simultaneously analyzed the samples through parallel experiments with classical and molecularly imprinted solid-phase extraction systems while detection was performed by liquid chromatography-tandem mass spectrometry (LC-MS-MS) measurements. They concluded that molecularly imprinted cartridges have a higher selectivity than the classical ones and might be used as complementary method for chronic users [7]. Figueiredo et al. reported a direct extraction and quantitation of benzodiazepines in human plasma by using MIPs. They utilized the electrospray ionization mass spectrometry (ESI-MS) as a detection platform with a high and selective extraction capability, ionic suppression and a short analysis time and a high analytical speed. They reported a linear calibration curve in the range of 10–250 μg/L (*r* > 0.98) with a low LOQ quantification < 10 μg/L that also had an acceptable precision and accuracy for day-to-day and in-day measurements [8]. Rezaei et al. utilized MIPs with ESI-ion mobility spectrometry (IMS) system for detection of primidone (an antiepileptic drug) from complex matrices such as pharmaceutical and human samples. They concluded that the combination of MIPs with ESI-IMS was a very sensitive analytical tool for selective extraction and detection of the target molecule due to its wide linear dynamic range, good recovery, and low relative standard deviation (RSD) of 0.02–2.00 μg/mL, above 90%, and below 3%, respectively [9].

Cannabinoids like marijuana and hashish are the most commonly used illicit drug [10,11,12]. Nestic et al. reported a combination of MIP integrated with gas chromatography (GC-MS) for simultaneous determination of tetrahydrocannabinol and its main metabolite in urine samples. They reported that the performance of the method completely meets the requirements of toxicological analysis although the extraction recovery, LOD and lower limit of quantification (LLOQ) only suggested performance comparable with the described method in the light of results they achieved [10]. Sanchez-Gonzalez et al. also reported a micro-solid extractor for cannabinoids for assessing plasma and urine analysis of marijuana abusers by the combination of MIPS with a HPLC-MS/MS system. They reported LOQ values for plasma and urine samples in the ranges of 0.36–0.49 ng/L and 0.47–0.57 ng/L, respectively, with an accurate method for inter-day and intra-day analytical recovery performances [11]. Cela-Perez et al. also reported water-compatible imprinted pills for a combined cannabinoids extraction/detection method in urine and oral fluid. They optimized the extraction performance by tuning the MIP composition with respect to screening results of a non-imprinted polymer library. They developed a linear method for urine and oral fluid in the ranges of 1–500 ng/mL and 0.75–500 ng/mL, respectively. They finally applied the developed method to four urine and five oral fluid samples in which low imprecision (lower than 15%) and varied recovery (50%–111%) and good process efficiency (15.4%–54.5%) were determined [12].

Opioids like heroin and opium are substances that act on the opioid receptors to produce morphine-like effects [13,14,15]. Andersson et al. reported one of first studies including morphine and endogeneous neuropeptide-imprinted polymers. They demonstrated a high binding affinity and selectivity in aqueous buffers which allowed study with biological materials. They also observed high binding constants (as low as 10^−7^ M) at levels of selectivity similar to those of antibodies. They concluded that the high binding affinities and selectivity could lead to the use to MIPS in enzyme- based assays like ELISA and immune-affinity techniques for isolation/separation of water-soluble biologically related compounds [13]. Piletska et al. also developed a multisensory method for drugs of abuse while optimizing the MIP composition by computational techniques. They reported an imprinting factor of 3 for morphine by comparing the recognition capabilities of imprinted and non-imprinted polymers [14]. Devanathan et al. reported a covalently imprinted polymer having subpicomolar binding affinity in an aqueous environment and a well-defined and homogeneously distributed cavity. They utilized plasmon-waveguide resonance spectroscopy as a sensitive optical detection of the target molecules, which allowed achieving the tightest binding ability (up to 10^3^-folds) in comparison to the competitor molecules [15].

Another class of the drugs often studied are stimulants like cocaine, amphetamine, and methamphetamine [14,16,17]. As mentioned before, Piletska et al. used a set of MIPs for detecting some drugs in combination with HPLC analysis. They reported the imprinting factors for cocaine, deoxyephedrine and methadone as 1.8-, 4.2-, 6-folds, respectively, under optimized conditions [14]. Li et al. reported a novel stimulant assay by combining colloidal crystals with MIPs for theophylline and ephedrine as template molecules. This approach led to a rapid, handy, sensitive and specific detection system due to its structural features such as highly ordered and interconnected macropores with thin hydrogel walls. Homogenously distributed nanocavities on the walls enabled a rapid, easy, sensitive and direct response during the molecular recognition process without any need forf transducers and analyte treatments. As concluded in the article, a synergetic contribution of structural features and MIPs results in an extremely high sensitivity at such an analyte concentration as 0.1 fM and specificity even in spiked urine samples [16]. Club drugs like methylenedioxymethamphetamine (MDMA), flunitrazepam, γ-hydroxybutyrate and dissociative drugs like ketamine, phencyclidine (PCP) and its analogs, *Salvia divinorum*, and dextromethorphan (DXM, found in some cough and cold medications) were extensively examined targets [14]. Djozan et al. reported a solvent-free and sensitive method for analyzing the methamphetamine, amphetamine and ecstasy levels in human urine. They combined inside-needle trap and MIPs while coating the internal surface of a hollow stainless steel needle with a MIP layer. Due to the fact there was no requirement for an extraction solvent, the method developed was quite fast and simple. They reported LOD, LOQ and relative recovery values of 12 ng/mL, 40 ng/mL and 81%–93%, respectively, with a low relative standard deviation of 4.9% for six repeated experiments [17].

Hallucinogens like lysergic acid diethylamide (LSD), mescaline, psilocybin (magic mushrooms) are another class of targets [18]. Chapuis-Hugon et al. followed a non-covalent imprinting approach to develop a selective extractor for LSD from hair and urine samples. For this aim, they performed offline extraction before HPLC-MS analysis and reported a successful detection of LSD at a low concentration of 0.1 ng/mg in hair samples with an extraction recovery of 82%. For urine samples, easy detection at only 0.5 ng/mL with extraction recovery of 83% was also reported [18].

The compounds including anabolic steroids, inhalants (solvents and gases), nicotine, and alcohol are other intensively examined targets [19,20,21,22,23,24,25]. Zhou et al. applied Pickering emulsion polymerization to synthesize MIPs for steroid recognition. They reported that the combination of hydrophobic and hydrogen-bond interactions that were located in imprinted cavities resulted in a high selectivity for similar steroid structures [19]. Tan et al. used MIP as a recognition coating on a quartz crystal thickness-shear mode sensor for nicotine detection in human serum and urine. They achieved a highly selective and sensitive response that was linear over a wide nicotine concentration range of 5.0 × 10^−8^–1.0 × 10^−4^ M with a detection limit of 2.5 × 10^−8^ M [20]. Krupadam et al. synthesized nicotine-imprinted nanocavities of 24.0 ± 5.0 nm size which were homogeneously distributed through the polymeric structure. The MIPs developed showed a high selectivity with a dissociation constant (Kd) around 10^−5^ M, similar to those of the natural analog (acetylcholine esterase). They concluded that MIP-based artificial receptors are very useful for isolating and separating water-soluble biologically related compounds and detecting nicotine levels for addicted patients [21]. Zhou et al. also synthesized nicotine-imprinted polymers via a reversible addition-fragmentation chain transfer polymerization. They were able to form monodispersed beads with an average size of 1.55 μm. They concluded that these beads could be utilized as building blocks for developing chemical sensors and polymer-enzyme conjugates for analytical applications [22]. Matsuguchi and Uno developed a quartz crystal microbalance (QCM)-based sensor for volatile organic compound for detection of xylene and toluene in the vapor phase. They concluded that the simplicity and reliability of the developed sensor had promising potential in forming sensors combined with a MIP although some further improvements were needed in light of selectivity and response time performance [23]. Yang et al. developed a MIP-based SPE for assaying hair nicotine levels in smokers and non-smokers to investigate exposure to environmental tobacco smoke. They reported LOD and LOQ values of 0.2 ng/mL and 0.5 ng/mL, respectively, while determining a wide linear concentration range of 0.5–80 ng/mL with a regression coefficient greater than 0.987. The nicotine levels determined in smokers and non-smokers varied between 5.1–69.5 ng/mg hair and 0.50–9.3 ng/mg hair, respectively [24]. Wu et al. developed an amperometric sensor for nicotine by combining titanium dioxide, a conductive polymer [poly(2,3-ethylenedioxythiophene), PEDOT] and an imprinting approach. They reported a linear detection range, LOD and imprinting factors of 0–5 mM, 4.9 μM, and 1.24, respectively. They also evaluated the sensitivity enhancement with respect to the electroactive surface area and at-rest stability over 3 days, in which the current response remained at around 85% of its initial value at the end of the second day [25].

**Figure 3:**
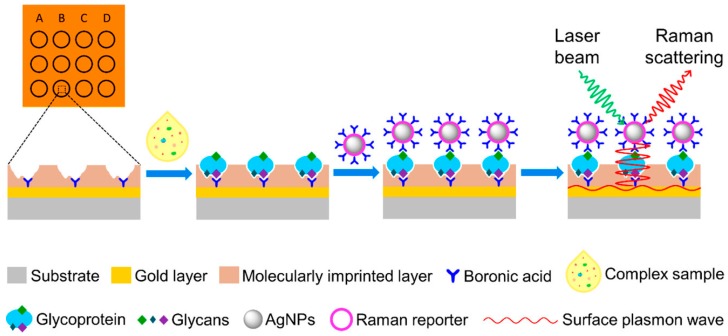
Schematic illustration of the MIP-based immunosandwich assay for recognizing the target (erythropoietin) glycoprotein. Reprinted with permission from [30]. Copyright (2016) American Chemical Society.

Forensic analysis approaches are also applicable for anti-doping purposes to determine doping with performance-enhancing drugs, stimulants, steroids, and corticosteroids [26,27,28,29,30]. Ozgur et al. developed a mass sensitive sensor for real time estradiol detection. They synthesized MIP nanoparticles and used them as recognition elements on quartz crystals. They reported that the resulting nanosensor had a high selectivity and sensitivity against target molecules in the concentration range of 3.67 nM–3.67 pM and the LOD and LOQ values they calculated were quite low, at 613 fM and 2.04 pM, respectively [27]. Zulfiqar et al. developed a MIP-SPE system for extracting and screening multiple steroids in urine. They analyzed a series of twelve structurally similar and commercially available compounds while imprinting only testosterone as a template. They reported effective LODs between 11.7 and 27.0 pg for individual steroids when investigating concentrations (equal for each steroid) between 0.234 and 0.540 ng/mL in urine. They also demonstrated multiple screening applications using a 10 ng/mL mixed sample [28]. Kellens et al. performed bulk and miniemulsion polymerization to produce colloidal particles, which were used for testosterone recognition. They compared the performances of bulk and colloidal MIPs and determined that the imprinting factor increased from 2.2 to 6.8 due to the smaller size, homogeneity and increased surface area of colloidal particles. They concluded that water-based stable MIP dispersions might be useful to construct sensing platforms via spin-coating or dropcasting methods [29]. Tu et al. developed a MIP-based plasmonic immunosandwich assay for erythropoietin recognition in human urine through surface-enhanced Raman scattering (SERS) measurements. They reported a specific detection level as low as 29 fM in a short analysis time of only 30 min in total. They also determined that the cross-reactivity of the assay varied in the range of 1.9%–9.6% for a 1000-fold higher concentration for interfering glycoproteins and non-glycoproteins and additionally it was only 0.8% for a 10,000-fold higher concentration of glucose [30] (Figure 3).

Poisons including cyanide, arsenic, nightshade, hemlock, curare, nicotine, caffeine, quinine, atropine, strychnine, and brucine were also intensively evaluated targets in the MIP literature [31,32,33,34]. Jackson et al. imprinted 2-aminothiazoline-4-carboxylic acid (ATCA), a chemically stable metabolite of cyanide, on the surface of a silica stir bar and used it for determining the endogenous level of ATCA in cases of cyanide poisoning. Without any derivatization requirements, the strategy enhanced the selectivity and sensitivity of ATCA detection in urine samples at a low concentration of around 400 ng/mL [31]. Liu et al. integrated electropolymerized MIPs with single-wall carbon nanotubes (SWNTs) for brucine detection in human serum. Linear concentration range, detection limit, and recoveries were determined as 6.2 × 10^−7^–1.2 × 10^−5^ M, 2.1 × 10^−7^ M, and 99.5%–103.2%, respectively [32]. Alizadeh et al. developed arsenic-imprinted nanoparticles for electrochemical ion detection. They inserted a hydrophobic chains (dodecanol) on the nanoparticles to improve durability, lifetime, and analytical characteristic of the polymeric membrane electrodes. They used the sensor for arsenic determination in different water samples and reported a wide concentration range of 5.0 × 10^−8^–1.0 × 10^−1^ M with a LOD value as 30 nM [33]. Nakamura et al. followed precipitation polymerization and multistep swelling and polymerization techniques to form monodisperse MIPs for strychnine recognition. They utilized liquid chromatography for separation and quantification purposes and evaluated the retention and molecular recognition performances of MIPs against not only the template (strychnine) but also some structurally relevant molecules, including brucine, quinine, quinidine, and indole. The retention factors and imprinting factors of strychnine were reported as 220 and 58 for the particles synthesized via precipitation polymerization, respectively, and 73 and 4.5 for the particles synthesized via multistep swelling and polymerization [34]. Xu et al. combined stimuli-responsive polymers with MIPs to synthesize dual (photonic and magnetic) responsive polymers for caffeine detection. They demonstrated that the recoveries ranged from 89.5% to 117.6% from real water and beverages after optimizing the adsorption/elution conditions while performing the experiment under UV (365 nm, adsorption) and visible light (release) [35].

*DNA:* Not only small molecules but also biomacromolecules, especially DNA, are supplying important information for forensic investigations. Although DNA sequences are 99.9% the same in every person, the remaining 0.01% is enough to distinguish one person from another, if you have the enough amount of DNA molecule to test [2]. Therefore, MIPs are very useful tools for the enrichment of DNA fragments from biological fluids [36,37,38,39]. Ogiso et al. used a MIP layer for electrophoretic DNA analysis and achieved the detection of target double-stranded DNA sequences in the presence of different sizes of interfering DNA fragments [36]. Diltemiz et al. developed a biomimicking sensor by creating thymine-imprinted cavities in MIPs with a synthesized adenine-based polymerizable monomer. They reported that the imprinted cavities were homogeneously distributed for thymine recognition with an affinity constant of 10 μM, whereas these sites interacted heterogeneously with uracil as a competitor nucleotide [37]. Ersöz et al. combined MIP-based SPE with a mass sensitive sensor for pre-concentration/detection of 8-hydroxy-2′-deoxyguanosine. They concluded that analytical performance of the proposed system was a promising alternative in comparison to electrophoresis [38]. Uzek et al. synthesized MIP-based monolithic cryogels for rapid plasmid DNA purification. They utilized hydrophobic interactions to recognize DNA molecules and directly integrated the developed column into a fast-protein liquid chromatography system [39].

**Figure 4:**
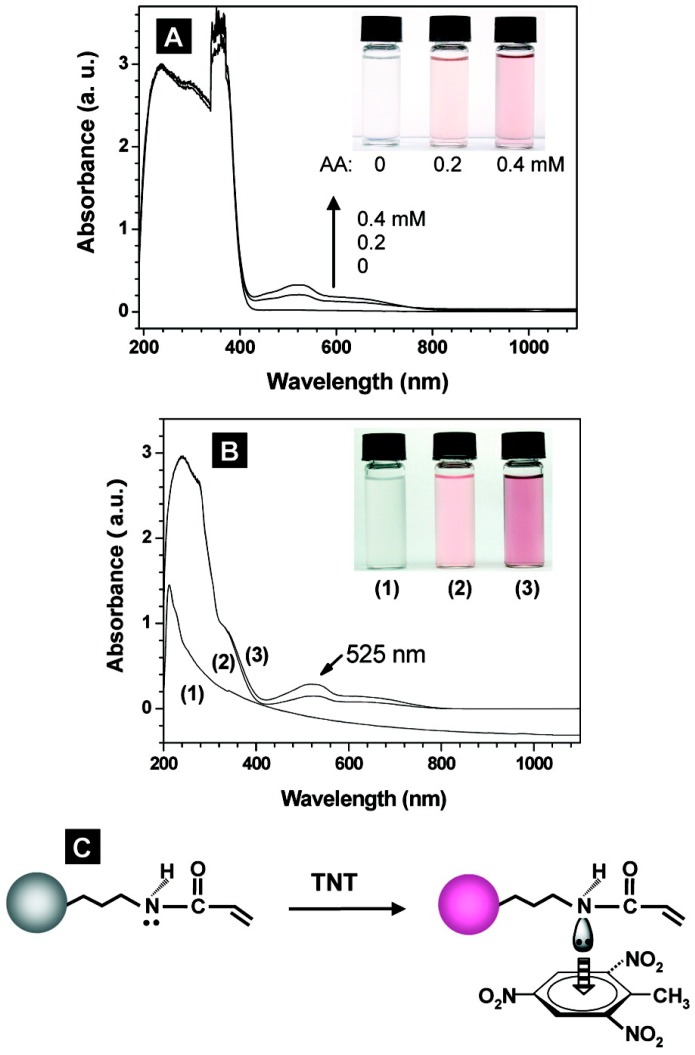
The interactions of TNT molecules with acrylamide and AA-APTS-silica nanoparticles. (**A**) The evolution of UV-visible spectra of TNT solution with increasing acrylamide amount (Inset colorful image shows the corresponding colors of TNT solutions); (**B**) The evolution of UV-visible spectra of AA-APTS-silica nanoparticles solution with increasing TNT amount: (1) without, (2) 0.25 and (3) 0.5 mM TNT (Inset colorful image shows the corresponding colors of nanoparticle solutions); (**C**) The schematic illustration for the charge-transfer complexing interactions between AA-APTS monolayer and TNT molecules. Reprinted with permission from [40]. Copyright (2007) American Chemical Society.

*Explosives*: Nitrobenzene, dinitrotoluene (DNT), trinitrotoluene (TNT), and cyclotrimethylene-trinitramine (RDX) were extensively studied explosives for criminal investigations [40,41,42,43,44,45,46,47,48,49]. Gao et al. synthesized core-shell imprinted particles via asurface functional monomer-directing strategy for selective 2,4,6-trinitrotoluene (TNT) detection. They used silica nanoparticles as a core material while acrylamide and ethylene glycol dimethacrylate were used as monomer and crosslinker, respectively. They also compared the performances of core-shell and traditional imprinted particles and concluded that core-shell MIPs have five times higher capacity and fast kinetics [40] (Figure 4).

**Figure 5:**
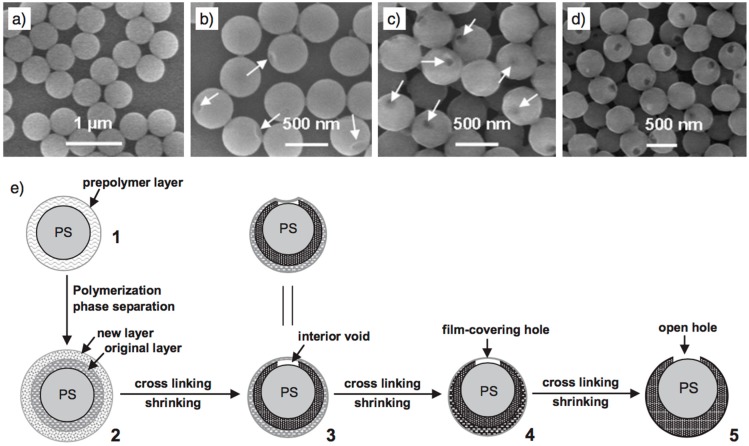
The formation mechanism of holes. (**a**–**d**) SEM images of four intermediate products with different polymerizing/cross-linking periods at a temperature of 60 °C: (**a**) Monodispersive core–shell microspheres (after 3 h); (**b**) core-shell microspheres with a dimple-like concave (after 6 h); (**c**) core-shell microspheres with a single hole covered with a layer of ultrathin film (after 12 h); and (**d**) core-shell microspheres with open holes (after 20 h); (**e**) Schematic illustration for the formation process of holes in the shells. The microphase separation and progressive volume shrinkage of shell materials leads to the formation of holes via five typical stages: (1) the formation of a prepolymer layer; (2) the microphase separation of two polymer shell layers; (3) the development of a small interior void at the shell; (4) formation of a film-covered hole; and (5) formation of an open hole in the polymer shells. Reprinted with permission from [41]. Copyright © 2007 WILEY-VCH Verlag GmbH & Co. KGaA, Weinheim, Germany.

Guan et al. developed hollow polymer with holes in the shells and core-shell microspheres for TNT detection. They reported the maximum TNT binding capacities of hollow and core-shell microspheres as 6.2 and 1.5 μmol of TNT, respectively, for single-runs of 20 mg microspheres [41] (Figure 5).

Li et al. evaluated the chemosensing performance of fluorescent conjugated MIPs for the detection of TNT and related nitroaromatic compounds in vapor phase. They demonstrated that vapor exposures for ten minutes caused a substantial decrease in fluorescence intensity and this behavior was repeated without any noticeable irreversible quenching [42]. Holthoff et al. reported a new and interesting strategy for integrating TNT-imprinted polymers into a surface enhanced Raman scattering platform. They deposited sol-gel derived xerogels on a SERS-active surface and achieved an apparent dissociation constant and detection limit for TNT of 23 μM and 3 μM, respectively [43]. Riskin et al. developed an ultrasensitive surface plasmon resonance (SPR) detection method for hexahydro-1,3,5-trinitro-1,3,5-triazine (RDX) by using gold nanoparticles crosslinked with bisaniline. They electrochemically polymerized gold nanoparticles in the presence of Kemp’s acid that allowed the selective and sensitive RDX detection with a quite low detection limit around 12 fM [44]. Riskin et al. followed similar strategy to develop a SPR sensor for pentaerythritol tetranitrate, nitroglycerin, and ethylene glycol dinitrate as well. They reported LOD values for pentaerythritol tetranitrate, nitroglycerin, and ethylene glycol dinitrate of 200 fM, 20 pM, and 400 fM, respectively [45]. Lordel et al. utilized MIPs as a selective extractor for the analysis of nitroaromatic explosives. They concluded that MIP-based extractors indicate a promising potential for miniaturized system due to a very large capacity value, higher than 3.2 mg/g [46,47]. Furthermore, they developed an online microextractor as well. By this way, they achieved a simultaneous extraction and determination of different nitroaromatic explosives with recovery values higher than 90% [48]. Mamo and Gonzalez-Rodriguez developed an electrochemical sensor for the detection of triacetone triperoxide (TATP) over a wide linear range of 82–44,300 μg/mL with a correlation coefficient of 0.996. They determined the LOD and LOQ values as 26.9 μg/L and 81.6 μg/L, respectively, with a quite good repeatability [49].

**Figure 6:**
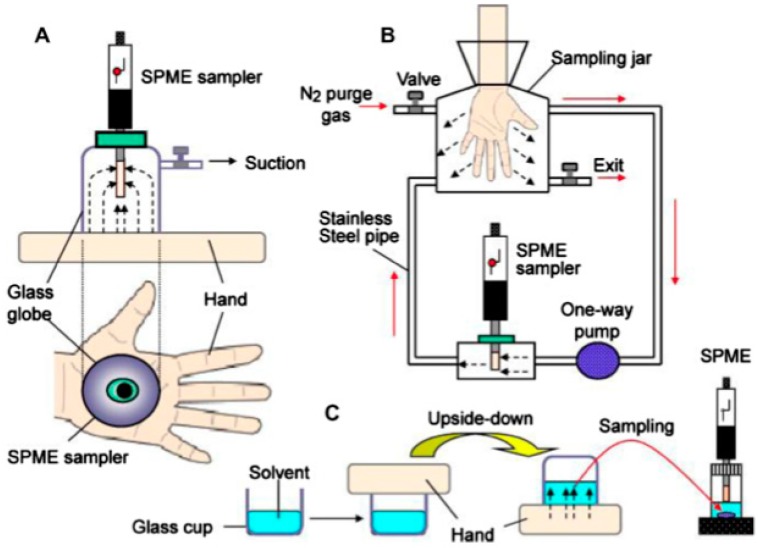
Systems for sampling volatile compounds emitted from the skin. (**A**) Direct SPME in sealed glass globes; (**B**) direct SPME in flow sampling chambers; and (**C**) liquid sampling in glass cup. Reprinted with permission from [53].

*Gunshot residues:* Components of gunpowder are not found in the general population, so the residues of gunshot on clothing or hands of a suspect are good indicator of a fired gun. Gunshot residues consist of inorganic (lead, antimony, barium, calcium, and silicon) and organic (diphenylamine, ethyl centralite and nitrodiphenylamine, dinitrotoluene, nitrobenzene) residues [50] (Figure 6). Studies show that there are at least 136 organic compounds like additives coolants, plasticizers, anti-wear additives that may contribute to gunshot residue [51]. Generally molecular imprinting methods are used for the sample preparation step before a chromatographic or mass spectroscopy analysis of gunshot residue analysis [52]. Pereira et al. developed MIPs for the retention of diphenylamine, one of the organic residues of gunshots, and demonstrated their capacity by HPLC and UV-visible spectroscopy measurements. They concluded that recognition of target molecules was really fast and reached a maximum retention in only the first five minutes [52].

*Fire accelerants:* Generally a molecular imprinting method is used for the sample preparation step before a chromatographic or mass spectroscopy analysis, in fire debris analysis for accelerant compounds like gasoline, kerosene and alcohol [53,54]. Kabir et al. summarized the recent advances in micro-sample preparation for forensic science while considering fire debris analysis and toxicology [53]. Alizadeh et al. developed an ethanol sensor by combining multi-walled carbon nanotubes, nano-sized MIPs, and poly(methyl methacrylate) as conducting element, recognition element, and adhesive substance, respectively. They achieved a reversible sensor response with a low relative standard error around 2.6%. They also reported that the sensor has a linear response in the concentration range of 0.65–45.0 ppm with a LOD value as 0.5 ppm. They concluded that the sensor response did not vary significantly within 4 months (confidence level = 95%) that indicated good durability and a long shelf-life [54].

*Chemical warfare agents:* In terms of national security and defense, the detection of chemical warfare agents is a very important target for forensic studies [55,56,57]. Boyd et al. developed a waveguide sensor for pinacolyl methylphosphonate (PMP, a hydrolysis product of the chemical warfare agent soman). They utilized a fluoropolymer with a refractive index of 1.29 that is slightly less than water (1.33), to develop lanthanide-based fluorescent detection of PMP. They concluded that the synergetic effect of MIP (selective and sensitive recognition) and fluoropolymer (inherent sensitivity and fast response time) allowed detecting the target molecules within seconds and had potential to use for warfare agent release at or below the time-weighted average/airborne exposure limit that is as low as parts-per-trillion range for the nerve agents [55]. Prathish et al. developed a potentiometric biosensor for the specific recognition of methylphosphonic acid (MPA), which is the degradation product of nerve agents such as sarin, soman, VX, etc. They plasticized MIPs with 2-nitrophenyloctyl ether on a polyvinyl chloride matrix. The sensor gave a linear response in the concentration range of 5 × 10^−5^–1 × 10^−1^ M with a LOD value as 5 × 10^−8^ M. They also reported that the sensor had a rapid response whereby 75% of the response was realized in 2 min and reached equilibrium in 5 min. They concluded that the sensor was stable, reusable, portable, and ready-to-use for in situ detection for not only for MPA, but also for actual chemical warfare agents and their simulants [56]. Lu et al. mentioned the importance and emergency of a real-time and on-site detection of chemical warfare agents due to the terrorist threats in their review article. They also summarized that MIPs serves as a promising potential to complete the requirements with their features including strong mechanical strength, flexibility, long-time storability, designing in required geometry and structure, and, of course, low cost [57].

*Environmental forensics*: Environmental forensic is the area of interest in which finding the source and age of an environmental contaminant like an oil spill, and heavy metal pollutants is investigated [58,59,60]. In terms of the legislative framework for environmental forensics, Mudge comprehensively summarized environmental forensics and the importance of source identification. Starting from national, regional and US legislations, the author mentioned the source identification methods and environmental forensic problems including illegal discharge, fugitive emissions or discharge, deliberate fly-tipping, historical discharges, and altered environmental processes. The author also compiled tools for source apportionments on the basis of chemical and biological approaches [58]. Davis et al. focused on determining release periods of petrochemicals to groundwater (in Whitehorse, Yukon) for use in geochemical forensics. In this article, they studied several forensic methods using a Geographic Information System (GIS)/Access^©^-based data visualization tool to investigate the source and timing of hydrocarbon releases at a site in Whitehorse, Yukon Territory [59]. Alizadeh et al. also applied a molecular imprinting approach to electrochemically detect TNT in different water and soil samples. The sensors worked with a dynamic linear range of 5 × 10^−9^–1 × 10^−6^ M and a low LOD value of 1.5 × 10^−9^ M [60].

### 3.2. Uses of MIPs for Pre-Concentration/Sample Preparation/Extraction

Molecular imprinted materials have a variety usage purposes like drug delivery, biomimetic enzyme catalysis, separation, extraction, and sensing [61,62,63,64,65,66,67]. In the area of forensic sciences, the most common usages are extraction, pre-concentration, and detection of target molecules from a variety of body samples and fluids like hair, saliva, urine, pericardial fluid, blood and questioned samples like chewing gums, cigarette butts [68].

**Figure 7:**
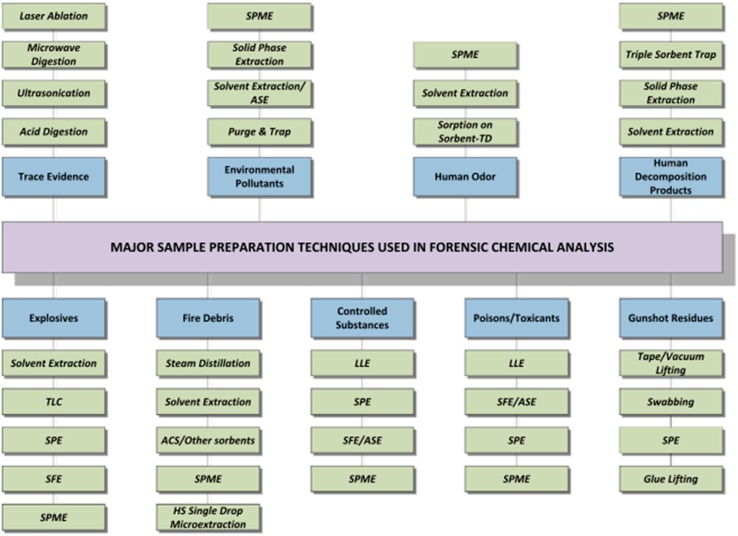
Major classes of forensic samples and their sample preparation techniques. Reprinted with permission from Ref. [53]. ACS: Activated carbon sorbents; ASE: accelerated solvent extraction; HS: Head space; LLE: Liquid-liquid extraction; SFE: supercritical fluid extraction; Sorbent-TD: Sorbent-Thermal desorption; SPE: Solid phase extraction; SPME: solid phase micro-extraction; TLC: Thin layer chromatography.

**Figure 8:**
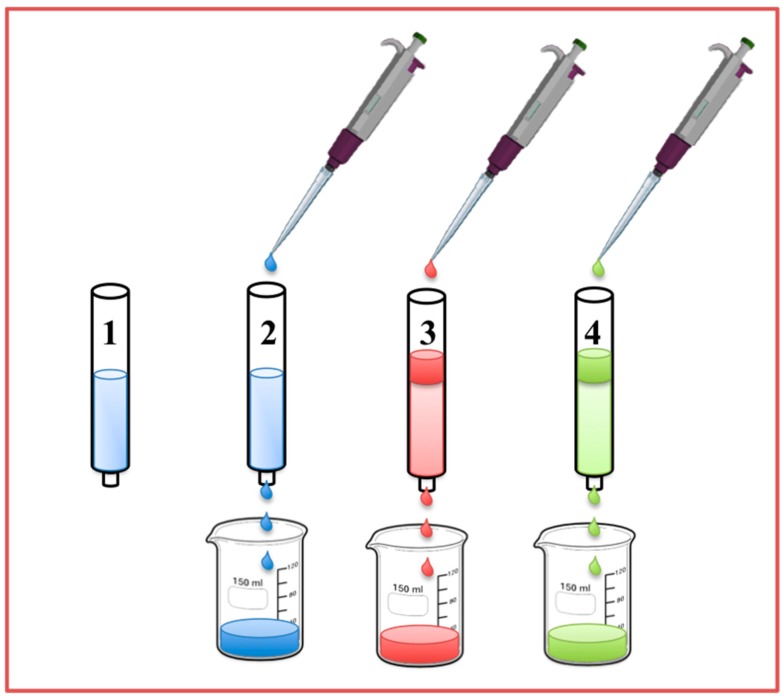
Schematic representation of off-line solid-phase extraction. 1. Equilibration of the column packed with MIP particle or monolith synthesized via in-situ polymerization; 2. Sample loading; 3. Washing out of interfering substances; 4. Elution of the analyte pre-concentrated.

Even though the advanced analytical technique is the main step for quantification of target molecules, a pre-concentration step is a necessity in some cases like trace analysis. Extraction has widely been used in different fields including for environmental, food, natural products, pharmaceuticals, toxic and forensic samples [69]. Kabir et al. comprehensively summarized the major classes of extraction methods used for forensic analysis [53] (Figure 7). The main purpose of extraction is generally pre-concentration of target molecules before the detection step without further separation steps. There are two main approaches followed for extraction step: off-line and on-line extraction [46,47,48]. In general, solid-phase extraction is applied as off-line pre-concentration just before the quantification of target molecules (Figure 8).

**Figure 9:**
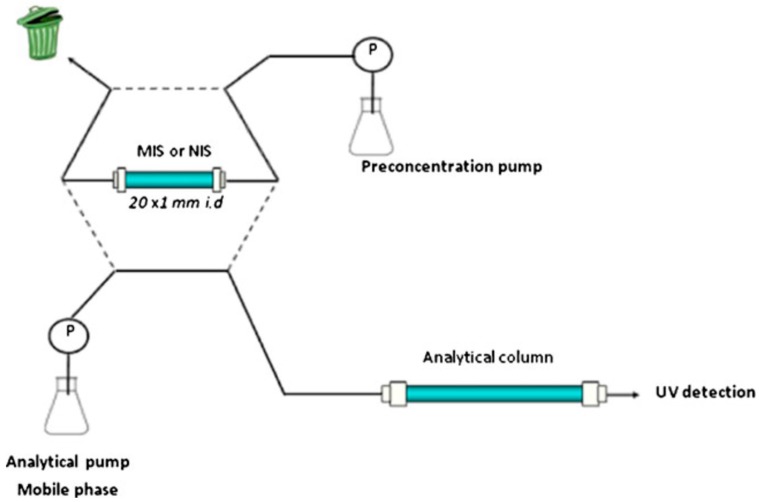
Scheme of the online coupling of the MIP-based extractor with HPLC. Reprinted with permission from [48]. Copyright © 2013 Springer International Publishing AG.

In this approach, the extraction column is filled with MIP particles or synthesized monolithic structures via in-situ polymerization and equilibrated with solvent. Then, the sample is flowed through the column. After washing out the interfering substances, the target molecules are eluted by using a desorbing agent at higher concentration. Although this approach is simple and intensively used, it is really time-consuming and does not allow the automation of the detection and it is hard to handle samples in higher amounts with this approach. Therefore, on-line extraction/pre-concentration has attracted researchers’ interests (Figure 9). In this approach, it is possible to attach a column to chromatographic system directly via some modifications of sampling step by using different and complicated valve-systems.

## 4. Instrumentation and Detection

**Figure 10:**
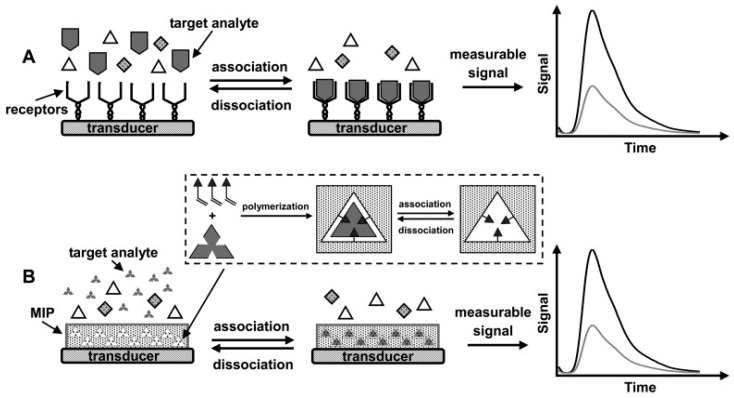
Schematic representations of (**A**) antibody-based chemosensor and (**B**) MIP-based biomimetic sensor. Inset in (**B**) shows the concept of molecular imprinting. Reprinted with permission from [70].

Sensitivity enhancement is one of the advantages of molecular imprinting technology. Basically, MIPs are used as recognition elements on the transducers instead of biorecognition element such as antibodies, enzymes, DNAs, and aptamers etc. (Figure 10). The integration of MIPs into commercial systems offers several advantages due to the different possible MIP-based designs while keeping all instrumentation and detection methods constant. In this part of the review, we summarize some of the interesting methods utilizing MIPs for forensic analysis.

### 4.1. Spectroscopy

**Figure 11:**
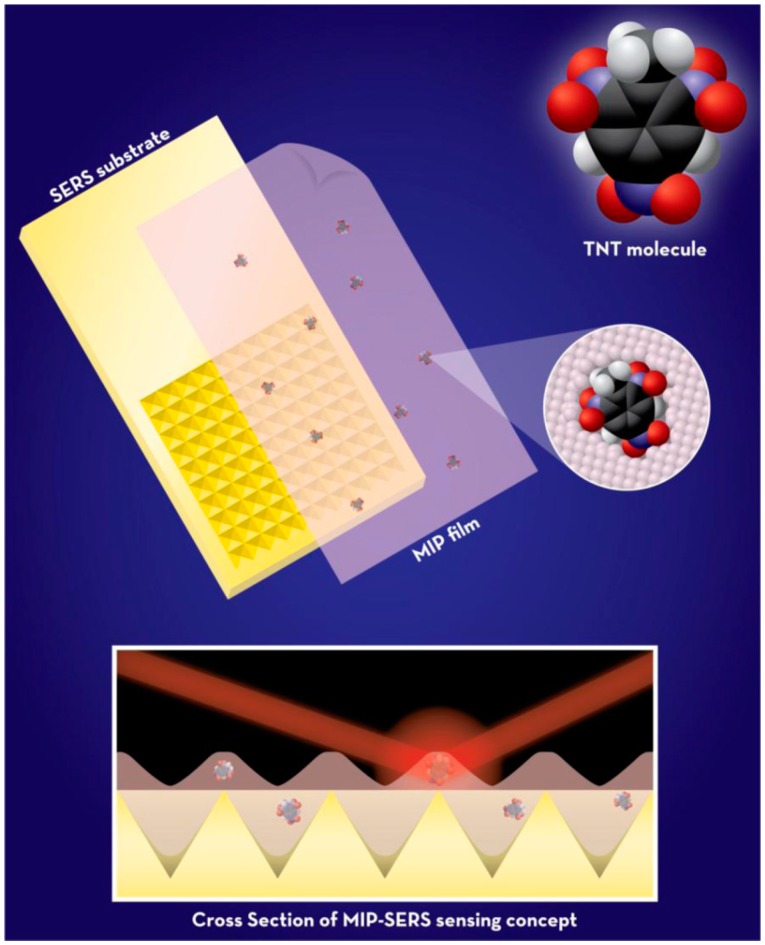
Xerogels based MIP-SERS detection concept. Reprinted with permission from Ref. [43].

*Surface-enhanced Raman scattering (SERS)* is an extremely sensitive spectroscopic technique based on molecular vibrations but it has some drawbacks, as any molecule with a similar chemical structure may interfere with the SERS and cause doubtful results. Thus, molecular imprinting provides a good solution to increase the selectivity. Holthoff et al. presented a novel strategy to combine molecular imprinting technology with SERS to improve the detection of explosives via deposing imprinted xerogels which provide a high porosity along with large surface areas on SERS active surface [43]. By using the attractive structural features of xerogels in combination of MIPs, they were able to develop a quite sensitive SERS substrate for TNT detection (Figure 11).

*Ion mobility spectrometry (IMS)* is a widely used analytical method used for detection and identification of trace amounts of vapors based on the mobility of gas phase ions in a weak electric field. IMS has many uses in the area of forensic sciences, including personal markers, and detection of explosives, toxic substances and narcotics [71]. There have been many studies for implementing the molecular imprinting technology to IMS for the detection of explosives and drugs in the last decade [72,73,74].

### 4.2. Optical Detection

*Surface plasmon resonance (SPR)* is an optical sensor based on optical excitation of surface electrons of metal on a metal-dielectric interface. SPR is a widely used method due to its availability for mobility, ease of modification and production of sensor surfaces available for multi-analyte detection via spotting and sensitivity [75].

**Figure 12:**
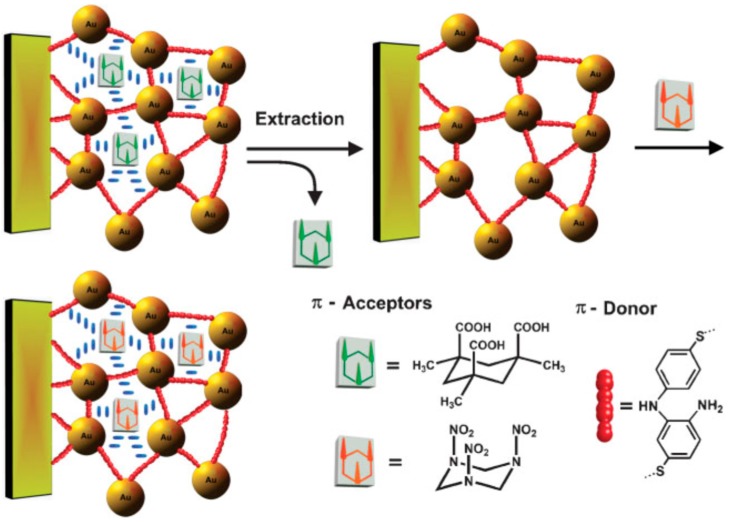
Imprinting of Kemp’s acid molecular recognition sites into the composite of bisaniline-crosslinked Au NPs associated with a Au electrode, for RDX analysis. Reprinted with permission from [44]. Copyright © 2010 WILEY-VCH Verlag GmbH & Co. KGaA, Weinheim, Germany.

Molecular imprinting-based SPR sensors are used for a variety of biological structures like proteins, antibodies and antibody fragments, hormones, cells, viruses, aptamers [76,77] as well as drugs, explosives, and toxins. Riskin et al. reported molecularly imprinted gold nanoparticles for the detection of various explosives. The produced imprinted nanoparticles had high affinities and selectivity toward the imprinted explosives with detection levels of 200 fM for pentaerythritol tetranitrate and 20 pM for nitroglycerin [45] (Figure 12).

### 4.3. Colorimetric Sensing

Photonic crystals are periodic nanostructures that affect the motion of photons that can also be used as label-free detecting platforms due to their structural coloration ability. Hu et al. demonstrated a method for combining photonic crystals with molecular imprinting technology via hierarchical porous structured molecularly-imprinted hydrogel for sensor applications [78]. They achieved an enhanced response by implementing the molecular imprinting strategies into photonic crystals which found applications in forensic sciences. Meng et al. showed a semi-quantitative method for atropine, an important alkaloid in forensics, via a molecularly imprinting-based photonic hydrogel with a very low LOD value of 1 pg/mL [79]. There are some other studies showing sensitive colorimetric detection for organophosphorus-based nerve agents like sarin, soman, VX and R-VX, explosives, ketamine and morphine [16,79,80,81,82].

*Chemiluminescence sensing* is the one of the more sensitive colorimetric sensing strategies. Han et al. developed a molecular imprinting-based electroluminescence sensor which has the possibility of becoming an alternative to a trained dog for the detection of hidden drugs in luggage, mail, vehicles, and aircraft, and in the human body [83]. They achieved detection of methamphetamine hydrochloride (MA) and morphine via the combination of molecularly imprinted sol-gel polymers with a light emitting material and a multi-walled carbon nanotube composite. A detection limit of 4.0 × 10^−15^ M was achieved. This method is quite promising for detection of hidden drugs or explosives from their odor. There are also molecular imprinted chemiluminescence sensors for poisons in the literature. Liu et al. developed a molecular imprinted chemiluminescence sensor for the determination of brucine, which is a dangerous poison. The detection limit was reported as 2.0 × 10^−9^ g/mL [84].

*Fluorescence sensing* is another colorimetric sensing approach for ultrasensitive target detection in combination with MIPs. Fluorescence emission may be obtained by a variety of fluorophore dyes or different-sized quantum dots. Fluorescent-labeled MIP materials could be used for visualizing the recognition between the imprinted material and the template molecules. Use of fluorescent probes in combination with MIPs has mainly focused on the detection of poisons and vapors of explosives and fire debris analysis [42,85,86,87].

### 4.4. Mass Detection

Piezoelectric (PZ) transduction sensors have many sensing applications for a variety of target structures. Their advantages in gas phase detection are quite important for on-site analysis. Quartz crystal microbalances, surface acoustic wave-guides, microcantilevers, and micro-electromechanical systems are among intensively studied mass sensitive sensor systems.

*Quartz crystal microbalance (QCM):* Chianella et al. reported a molecular complete imprinting solution to detect microcystin-LR, a cyanobacteria-based toxin [88]. They prepared both MIP-based solid phase extraction cartridges for pre-concentration, and a MIP-based QCM sensor. They achieved a limit of detection of 0.35 nM of toxin.

*Surface/bulk acoustic wave:* Surface acoustic wave (SAW)- and/or bulk acoustic wave (BAW)-based sensors work according to the variation of velocity of the acoustic waves based on the increased mass on the transducer. Percival et al. studied a molecular imprinting-based surface acoustic wave sensor for the detection of an anabolic steroid, nandrolone. They synthesized MIP layers via a covalent imprinting approach and reported a frequency shift of up to 0.2 ppm with the sensor preferring the target, nandrolone, to analogous compounds. They also concluded that such acoustic wave devices could be integrated into lightweight and low cost oscillator circuits allowing inexpensive screening technique to be developed [89]. Pan et al. developed a novel molecular imprinting-based SAW sensor to the detect warfare agent VX. The detection limit of the produced sensor was reported as 0.15 mg/m^3^ and after 18 months, its detection signal decreased by about only 4.4% [90].

Even though caffeine is a widely used legal natural stimulant, it has also importance for forensic purposes. Tanada et al. reported caffeine analysis can be used for forensic hair discrimination [91]. Liang et al. prepared a biomimic BAW sensor by coating the surface with caffeine-imprinted polymers. The sensor was highly selective and gave a sensitive response in the linear concentration range of 5.0 × 10^−9^–1.0 × 10^−4^ M at pH 8.0 with a LOD value of 5.0 × 10^−9^ M and high recoveries between 96.1% and 105.6% [92].

Tan et al. produced a molecular imprinting-based BAW sensor for the detection of paracetamol, a frequently encountered drug in suicide cases [20]. They reported the produced sensor was successfully used for the determination of paracetamol in human serum and urine with a limit of detection of 5.0 × 10^−3^ μM.

*Microcantilevers:* Use of atomic force microscopes (AFMs) for chemical and biological sensing is a trending area. A modified AFM microcantilever AFM was used as mechanical transducer via the increased mass on the modified cantilever with binding of complementary species. This method may be seen as an artificial nose due to the availability of odor detection at ultra-trace levels. Implementing microcantilever-based detection with molecular imprinting is a relatively new area of study [93].

*Micro-electromechanical systems (MEMS):* MEMS are made up of components between 1 and 100 micrometers in size for miniaturized sensors, actuators, and structures. The use of molecular imprinting technology is a relatively new area for this approach as well. Holthoff et al. developed a microbeam-based MEMS gas sensor with molecular imprinting for the detection of TNT and dimethyl methylphosphonate (DMMP), a simulant for the nerve gas sarin [43]. To form xerogel- based TNT and DMMP imprinted films on MEMS devices, complex mixtures containing TNT and DMMP were spin casted on MEMS devices.

### 4.5. Electrochemical Detection

Electrochemical sensors are a main sensor platform due to their simplicity, cost-efficiency and widely usability. Triacetone triperoxide (TATP) is one of the most common components of explosives and therefore Mamo et al. produced a highly sensitive electrochemical sensor for TATP via a differential pulse voltammetry-based molecular imprinted sensor. They produced a MIP-based glassy carbon electrode which demonstrated good performance at low concentrations for a linear and wide concentration range of 82–44,300 parts per billion (ppb) with a high correlation coefficient of R^2^ = 0.996. LOD and LOQ values were reported as 26.9 and 81.6 ppb. They also showed very good repeatability with precision values (*n* = 6, expressed as relative standard deviation (RSD)%) of 1.098% and 0.55% for 1108 and 2216 ppb, respectively. They concluded that the sensor can selectively detect TATP in presence of other explosives including pentaerythritol tetranitrate, 1,3,5-trinitroperhydro-1,3,5-triazine, octahydro-1,3,5,7-tetranitro-1,3,5,7-tetrazocine, and 2,4,6-trinitrotoluene [49].

### 4.6. Chromatography

There are many studies on MIP-based chromatographic methods in the area of forensic sciences like determining gunshot residues from hand and clothes. These residues are obtained by application of adhesive tapes to suspected person hands and clothes which may interfere with the results due to the adhesives on the tapes. Pereira et al. produced molecularly imprinted polymers for diphenylamine removal from organic gunshot residues, which is one of the most common components of gunshot residue [52].

**Figure 13:**
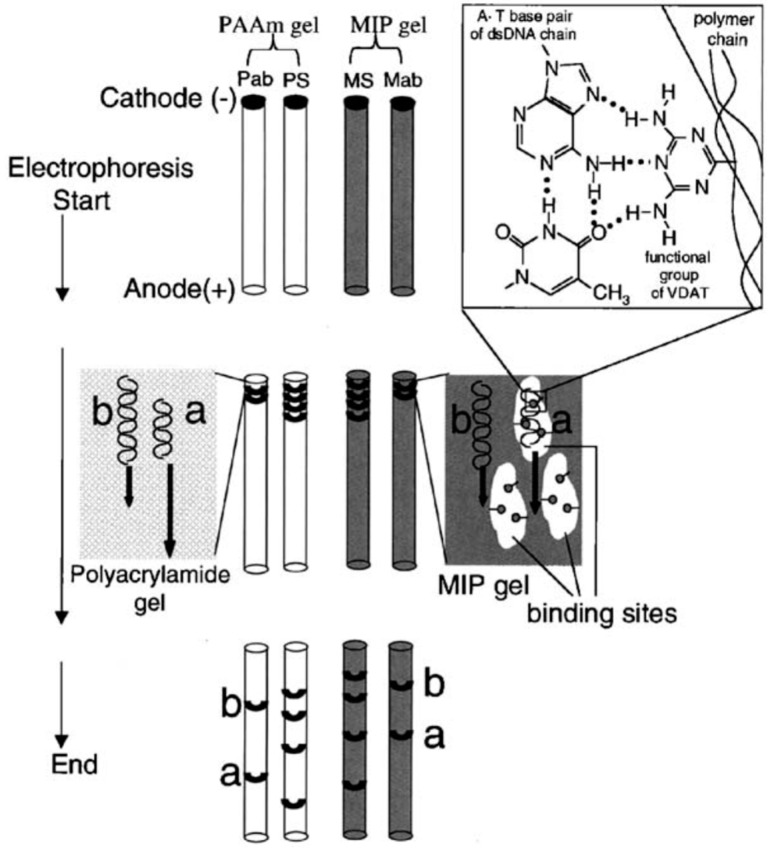
Principle of detection of the target dsDNA sequence by the dsDNA-imprinted polymer (MIP) gel in gel electrophoresis. A mixture of target dsDNA (**a**) and nontarget dsDNA (**b**) and the DNA standard size marker (S) is subjected to tube-gel electrophoresis with polyacrylamide gel (Pab and PS, respectively) and MIP gel (Mab and MS, respectively). During electrophoresis, migration of both nontarget (**b**) and target (**a**) dsDNA in polyacrylamide gel is dependent on the dsDNA fragment size. In the case of MIP gel, the migration of the target dsDNA (**a**) is dependent on both the fragment size and the capture effect of binding sites in the MIP gel. Consequently, the fragment size of the target dsDNA in the MIP gel should be larger than that in the polyacrylamide gel. While, the migration of the nontarget dsDNA (**b**) in MIP gel is dependent on the dsDNA fragment size. The fragment size of the nontarget dsDNA in the MIP gel should be the same as that in the polyacrylamide gel. Lengths of bold arrows indicate the velocity of dsDNA under the gradient of the electric field. The right top portion of the figure illustrates the binding site (imprinted cavity) of the target dsDNA in the MIP gel. Reprinted with permission from [36].

*Capillary electrophoresis (CE)* is a widely used method for trace analysis and it is a valuable tool in the area of forensic chemistry for analyzing inks, dyes, gunshot/explosive residues and drugs. Deng et al. produced a fiber-based SPME method by using molecular imprinting in capillary electrophoresis application for ephedrine and pseudoephedrine. Even though ephedrine and pseudoephedrine are therapeutic drugs, their amphetamine-like effects at high doses makes them important for forensic purposes. Limits of detection values were increased from 0.20 to 0.00096 μg/mL for ephedrine and 0.12 to 0.0011 μg/mL for pseudoephedrine via successful molecular imprinting-based SPME [94]. As mentioned before, Ogiso et al. utilized a CE system for selective DNA sequencing (Figure 13) [36]. They applied a mixture of target double-stranded (ds) DNA, non-target ds-DNA and standard DNA marker to capillary columns, imprinted and non-imprinted. The relative migration through the column was based on the size of the DNA as well as the selective interaction ability with polymer fillers (imprinted or non-imprinted). The place and length of the band indicated the sequence of the DNA fragments analyzed.

**Figure 14:**
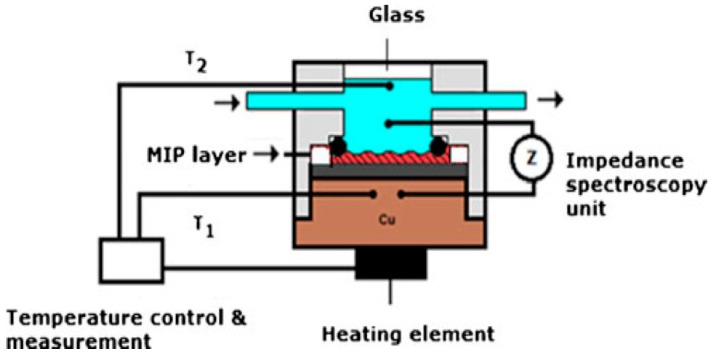
Schematic illustration of the general concept of the measuring setup. The temperature of the copper block, T1, is strictly controlled at 37.00 ± 0.02 °C. The heat flows from the copper block through the MIP layer to the liquid, where T2 is measured. Simultaneously with the temperature, the impedance is monitored. Reprinted with permission from [95].

*Heat transfer methods* are one of the recent approaches to develop selective biosensors in combination of MIPs. Peters et al. presented a novel approach for molecular imprinting-based nicotine detection from saliva via differential heat transfer resistance [95]. With the use of this method temperature differences via adsorption-desorption processes were measured precisely with the use of thermistor devices and they applied impedance spectroscopy to validate the results (Figure 14). They reported quite good dose-response results in the low concentration range of 0.2–0.75 μM with a high correlation coefficient (R^2^) of 0.97. LOD value reported as 125 nM was proper for biological samples in which nicotine levels vary in the range of 0–500 μM. Due to the importance of detecting small molecules for forensic purposes, this method offers a promising alternative.

## 5. Conclusions and Future Aspects

In this review, we aimed to draw a projection on the use of molecular imprinting in the related areas of forensic science. There are two main challenges with forensic science applications: (i) the complexity of the analytes and (ii) a quite low concentration of the analyte in this complex medium. Therefore, MIPs-based materials are intensively utilized as pre-concentrators before quantification through commercial techniques. Meanwhile, the integration of MIPs into traditional setups has recently attracted the efforts of the researchers (Table 1). Online pre-concentration, enhanced detection, improved selectivity and specificity, and excellent quantification limits as well as ease-of-production, robustness, cost-efficiency, and a variety of production strategies of MIP-based platform make them a promising alternative for achieving these aims and overcoming the mentioned drawbacks. Also, their other advantages such as chemical/physical stability, excellent compatibility with both of organic and aqueous media, reusability, long shelf-life and recognition capabilities make MIP-based design quite suitable for both sensing and sample preparation/pre-concentration of forensic targets including drugs, fire debris residues, explosives and gunshot residues, and chemical warfare agents. According to the reviewed literature reviewed, the attempts on MIP-based sample preparation/pre-concentration methods are much more closer to commercial products in respect to the sensory applications. Even though molecular imprinting-based materials have a variety of applications in many different areas, the evaluation of molecular imprinting-based sensor and sample preparation platforms should be expanded to the demands of the forensic sciences. Some of the recent studies have utilized computational approaches for determining the composition of imprinting materials, which would make possible to implement computationally well-designed systems to be used as effective, time saving and useful tools in the area of molecular imprinting. As a conclusion, MIP-based systems are rapidly and continuously growing platforms and their application in forensic science is at the beginning stages yet, demanding many novel designs.

## Figures and Tables

**Table 1:** An overview of the literature mentioned in this review in respect to device, method, materials, analyte, media and attributions.

	Combined Devices	Methods	Imprinting Materials	Analyte	Media	Attributes	Ref.
Legal/illicit drugs	LC-MS-MS	SPE	MAA and EDMA based monolith	Diazepam	Hair	LOD: 0.09	[6,7]
	EI-MS	SPE	MAA and EDMA based monolith	Benzodiazepines	Plasma	LOQ: <10 µg/L	[8]
	Electrochemical sensor	Composite NPs	PPyr sol-gel gold nanoparticles	Lorazepam	Artificial soln.	LOD: 0.09 nM	[9]
	GC-MS	SPE	MAA and EDMA based monolith	Δ9-THC-OH	Urine	LOD: 2.5–1 ng/mL	[10]
	LC-MS	SPE	EDMA and DVB based membrane	Cannabinoids	Plasma and urine	LOQ: 0.36 ng/L	[11]
	LC-MS-MS	SPE	AAm and EDMA based pill	Cannabinoids	Urine and OF	LOD: 0.75 ng/mL	[12]
	GC-FID	SPDE	MIP coated hollow stainless steel needle	MAMP, AMP, MDMA	Urine, saliva, hair	LOD: 12 ng/mL	[17]
	LC-MS	SPE	Ground and sieved MAA based monolith	LSD	Biological fluids	LOQ: of 0.2 pg/mL	[18]
	SERS	PISA	Boronate affinity-based AuNPs	Erythropoietin	Urine	LOD: 2.9 × 10^−14^ M	[30]
	IMS	SPE	MAA and EDMA based column material	Metronidazole	Human serum	LOD: 10 μg/L	[72]
	Optical response without instrument	Chemosensing	Molecularly imprinted photonic hydrogels	ATR, MOR	Biological samples	LOD: 1 pg/mL (ATR) 0.1 ng/mL (MOR)	[79,81]
Poisons	Quartz crystal-TSM sensor	Chemosensing	Imprinted polymer coating	Nicotine	Human serum and urine	LOD: 2.5 × 10^−8^ M	[20]
	Electrochemical sensor	LSV	PPDA/SWNTs composite film	Brucine	Human serum	LOD: 2.1 × 10^−7^ M	[32]
	Electrochemical sensor	EIS	MAA and EDMA based NPs	Arsenic	Biological fluids	LOD: 5.0 × 10^−7^	[33]
	Magnets and UV light	Magnetic response	Oleic acid modified Fe_3_O_4_ and Photoswitchable monomer	Caffeine	Water and beverage samples	Fast rebinding kinetics and high selectivity	[35]
DNA	Electrophoresis	GE	MIP gel as an electrophoretic matrix	Double strand DNA	Mixed-DNA sample	Simple and cost-effective detection	[36]
	No instrument	Adsorption	Hydrophobic cryogels	Plasmid DNA	*E. coli* lysate	Q: 45.31 mg DNA/g	[39]
Explosives and gunshot residues	SERS	Xerogels	Spin casted xerogel films	TNT	Artificial soln.	LOD: 3 µM	[43]
	SPR	Optical response	Au-NPs based composite	RDX, PETN, NG, EGDN	Artificial soln.	LOD: 12 fM–20 pM	[44,45]
	RP-LC	Online SPE	Sol-Gel organosilane	Nitroaromatic explosives	Post-blast samples	Extraction recoveries higher than 90%	[46,48]
	HPLC	SPE	Polymeric microparticles	Diphenylamine (DPA)	Gunshot residues	Retention up to 90%	[52]
	IMS	SPE	AAm and EDMA based particles	Nitroaromatics	Surface water	On-site detection	[74]
Fire accelerants	Chemiresistor sensor	Chemosensing	MWCNs-PMMA composites	Ethanol vapor	Air	LOD: 0.5 ppm	[54]
	QCM	Mass sensitive	PMMA and DVB based particles	Xylene and toluene	Air	Simple and reliable	[23]
Warfare agents	Electrochemical sensor	Potensiometry	MMA, VP and EDMA based particles	MPA	Natural water	5 × 10^−8^ M	[56]
	Fluorescence spectrometer	Fluorescence detection	Molecularly imprinted silica particles	Ricin	Artificial soln.	2–10 μM range	[85]

AAm: Acrylamide; AMP: Amphetamine; Atropine: ATR; AuNPs: Gold nanoparticles; DVB: Divinyl benzene; EDMA: Ethylene glycol dimethacrylate; EGDN: Ethylene glycol dinitrate; EI-MS: Electrospray ionization mass spectrometry; EIS: Electrochemical impedance spectroscopy; GC-FID: Gas chromatograpy-flame ionization detector; GC-MS: Gas chromatography-mass spectrometry; GE: Gel electrophoresis; IMS: Ion mobility spectrometry; LC-MS-MS: Liquid chromatography-mass spectrometry-mass spectrometry; LC-MS: Liquid chromatography-mass spectrometry; LSV: Linear sweep voltammetry; MAA: Methacrylic acid; MAMP: Methamphetamine; MDMA: Ecstasy; Morphine: MOR; MPA: Methylphosphonic acid; MWCNs: Multi-walled carbon nanotubes; NG: Nitroglycerin; NPs: nanoparticles; OF: Oral fluid; PETN: Pentaerythritol tetranitrate; PISA: Plasmonic immunosandwich assay; PMMA: Poly(methyl methacrylate); PPDA: Poly-o-phenylenediamine; PPyr: Polypyrrole; Q: Maximum adsorption capacity; QCM: Quartz crystal microbalance; RDX: Hexahydro-1,3,5-trinitro-1,3,5-triazine; RP: Reversed phase; SERS: Surface-enhanced Raman scattering; Soln.: Solutions; SPDE: Solid phase dynamic extraction; SPE: Solid-phase extraction; SPR: Surface plasmon resonance; SWNTs: Single walled carbon nanotubes; TNT: 2,4,6-trinitrotoluene; TSM: Thickness-shear-mode; VP: 4-Vinylpyridine; Δ9-THC-OH: Δ9-tetrahydrocannabinol.
